# Assessing Compassion in Korean Population: Psychometric Properties of the Korean Version of Sussex-Oxford Compassion Scales

**DOI:** 10.3389/fpsyg.2021.744481

**Published:** 2021-10-11

**Authors:** Jiyoung Kim, Jang-Won Seo

**Affiliations:** Department of Psychology, Jeonbuk National University, Jeonju, South Korea

**Keywords:** compassion, self-compassion, SOCS-O, SOCS-S, validity

## Abstract

A newly developed scale, the Sussex-Oxford Compassion Scale (SOCS) measures compassion for others and the self-based on an empirically supported five-elements definition of compassion: (a) recognizing suffering; (b) understanding the universality of suffering; (c) feeling for the person suffering; (d) tolerating uncomfortable feelings; and (e) motivation to act/acting to alleviate suffering. This study aimed to validate a Korean version of SOCS in a Korean adult sample. We administered the Sussex-Oxford Compassion Scale for Others (SOCS-O), the Sussex-Oxford Compassion Scale for the Self (SOCS-S), and other self-report measures of mindfulness, self-compassion, compassionate love, wellbeing, interpersonal reactivity, and mental health problems to analyze their psychometric properties. The findings support the five-factor hierarchical structure for the SOCS-O and SOCS-S, and as well as both scales’ adequate psychometric properties of measurement invariance, interpretability, internal consistency, floor/ceiling effects, and convergent/discriminant validity.

## Introduction

Compassion is not a new concept and has been discussed as a core human virtue by contemplative and religious traditions for thousands of years (Kirby et al., 2017; Gu et al., 2020). What is noticeable is the rapid burgeoning of interest in compassion toward self and others throughout many sectors of society, not only in the scientific community, but also in healthcare, education, and the justice system (Gilbert, 2014; Gu et al., 2020). In the healthcare system, the impact of compassion fatigue on the job performance of healthcare professionals has been extensively studied. Compassion fatigue which was initially referred to as secondary traumatic stress is associated with turnover intention and burnout (Sung et al., 2012). In the psychological realm, compassion is deeply ingrained in the underlying philosophy of psychotherapy. For instance, “unconditional positive regard” emphasized by Carl Rogers (Rogers, 1961), and the “capacity for concern” which lies at the heart of object relations theory points to the relevance of compassion to mental healthcare (Spandler and Stickley, 2011). In addition, compassion has been demonstrated to be associated with individuals’ positive physiological (Fredrickson et al., 2013) and psychological outcomes, such as adaptive emotion regulation (MacBeth and Gumley, 2012), greater wellbeing (Davidson and Schuyler, 2015), happiness (Mongrain et al., 2011), and reduced depressive symptoms (López et al., 2018). Indeed, a recent longitudinal study over 15years demonstrated stable effects of compassion for others on one’s wellbeing by evidencing the relation between high compassion with subjective perception of higher social support, life satisfaction, subjective health, and optimism (Saarinen et al., 2019).

Since Neff (2003b) operationalized the construct of self-compassion, it has gained popularity, and research on compassion toward self has become a trend integrating the construct of mindfulness with psychological approaches (Kyeong, 2013). Self-compassion, which consists of self-kindness, feelings of common humanity, and mindfulness, is a powerful predictor of adaptive characteristics, such as self-acceptance, life satisfaction, social connectedness, self-esteem, autonomy, and environmental mastery (Neff, 2003b). Also, it has been demonstrated to have negative associations with self-criticism, depression, anxiety, rumination, thought suppression, and neurotic perfectionism (Kirkpatrick, 2005; Kyeong, 2013). Indeed, a recent meta-analysis across 20 studies observed a large effect size for the relationship between self-compassion and psychopathology (MacBeth and Gumley, 2012). This result demonstrated that higher levels of self-compassion are associated with lower levels of symptoms of psychopathology, such as depression, anxiety, and stress.

The observed associations between compassion and mental health symptoms provided empirical support for the importance of compassion in enhancing psychological wellbeing, reducing negative repercussions of negative life events, and increasing resilience to stress (MacBeth and Gumley, 2012). Based on theoretical models that emphasize the robust importance of compassion, several interventions have been developed to cultivate compassion. Kirby et al.’s (2017) meta-analysis suggests that there are at least six empirically supported compassion-based interventions, including the following: Compassion-Focused Therapy (CFT; Gilbert, 2014); Mindful Self-Compassion (MSC; Germer and Neff, 2013); and Compassion Cultivation Training (CCT; Jazaieri et al., 2013). As is suggested, current evidence highlights the potential benefits of compassion-based intervention and the demand for further research into compassion is clear. However, there is still a lack of consensus on the key defining features of compassion. In the following sections, we will introduce varied conceptualizations of the two types of compassion. In addition, the empirically supported five-element definition of compassion, on which the Sussex-Oxford Compassion Scale (SOCS) is based, will be explained.

Goetz et al. (2010) conceptualize compassion as an affective state that arises from witnessing another’s suffering and having a desire to help. This definition differentiates compassion from empathy, which is the vicarious experience of emotion in others (Lazarus, 1991). Applying the definition of compassion of the Dalai (1995), CFT (Gilbert, 2014) describes compassion as involving two aspects: engagement, which is the sensitivity to distress in others and the self; action, which is described as commitment to alleviating suffering and preventing it. CCT developed by Geshe Thupten Jinpa elucidates compassion as a multidimensional construct consisting of cognitive, affective, intentional, and motivational components: (a) an awareness of suffering; (b) sympathetic concern linked to being emotionally affected by suffering; (c) a desire to see the relieving of the suffering; and (d) a response or a willingness to assist in alleviating the suffering (Jazaieri et al., 2013). Kanov et al. (2004) suggest that compassion consists of noticing, feeling, and responding. Others highlight common humanity and an understanding that the suffering is a shared experience (Feldman and Kuyken, 2011). Recently, in an attempt to consolidate varied conceptualizations into a comprehensive definition, Strauss et al. (2016) reviewed existing theoretical definitions of compassion and proposed that compassion consists of five elements: (a) recognizing suffering; (b) understanding the universality of suffering in human experience; (c) being empathetic to the person suffering and showing emotional resonance with the distress; (d) tolerance to uncomfortable feelings aroused in response to the suffering and the acceptance of the person suffering; and (e) motivation to act/acting to relieve suffering (Strauss et al., 2016). This five-element definition of compassion has received empirical support in Gu et al. (2017)’s factor analytic study.

In addition, Strauss et al. (2016) also systematically reviewed existing self-report and other-observed measures of compassion and concluded that nine questionnaires they reviewed (e.g., CCAT, Compassionate Care Assessment Tool; CLS, Compassionate Love Scale; SCBCS, Santa Clara Brief Compassion Scale; and SCS, Self-Compassion Scale) lack validity and reliability. Some of the measures fail to appropriately assess comprehensive aspects of compassion by including items phrased in contradiction to the response scale, they contain the word “compassion,” they are drawn from related concepts, such as empathy and have poor internal consistency and an insufficiently supported factor structure (Strauss et al., 2016). Continued use of these measures may significantly hamper progress in the scientific investigation and practice of compassion, as failure to grasp the full picture of compassion could lead to invalid research findings. This emphasizes the need for new measures assessing comprehensive aspects of compassion with robust psychometric properties. In response to this need, Gu et al. (2020) developed the Sussex-Oxford Compassion for Others Scale (SOCS-O) and the Sussex-Oxford compassion for the Self (SOCS-S). SOCS measures compassion for others and compassion toward the self with the empirically supported five-elements definition of compassion. Also, considering that compassion is identically processed whether it is directed toward the self or others (Feldman and Kuyken, 2011; Gilbert, 2014), it applies the same facets and factor structure for SOCS-O and SOCS-S. To support psychometric properties, they examined factor structure, interpretability, internal consistency, floor/ceiling effects, and convergent/discriminant validity with samples of 1,319 healthcare staff and 371 university students. For both scales, the findings supported the five-factor hierarchical structure, robust internal consistency and validity, and interpretability, and did not show floor/ceiling effects (Gu et al., 2020).

Compassion is a social mentality that could be shaped by various social contexts (Gilbert, 2014). In fact, recognizing and understanding the distress of others and the self, along with motivation to alleviate the suffering, require social interactions. This indicates that compassion may be experienced differently across collectivist and individualistic cultures, which are distinct in how people define themselves and their relations with others (Markus and Kitayama, 1991). In fact, a recent study conducted with independent samples of Australians and Singaporeans suggests that collectivist cultural norms may interfere with the expression of compassion toward others but facilitate self-compassion as compared to individualistic cultures, which places a high emphasis on self-actualization (Steindl et al., 2020). In addition, a study conducted with Japanese and American samples demonstrates that the associations between the two types of compassion and wellbeing and psychopathology appear different across the two cultures (Arimitsu et al., 2018). These findings may suggest that compassion may be differently operationalized across cultures. Therefore, we aim to develop a Korean version of SOCS and examine whether the same factor structure would be found with a Korean sample. In addition, we aim to analyze its invariance across gender, interpretability, reliability, floor/ceiling effects, and convergent/discriminant validity to support its psychometric properties. In accordance with the previous conclusions of Gu et al. (2020), we predicted positive significant associations between compassion for others and compassionate love, empathic concern, and perspective taking. In a similar regard, the two measures assessing self-compassion (i.e., SOCS-S and SCS) were expected to exhibit a strong positive correlation. Additionally, we expected positive correlations between self-compassion, mindfulness, and wellbeing, and negative relationships with personal distress, anxiety, and depression. Lastly, significant positive association between compassion for others and the self was predicted, but consistent with previous empirical findings (e.g., Neff and Pommier, 2013; Gu et al., 2020), we expected the two forms of compassion to be distinct. If the SOCS-O and SOCS-S measure distinguishable constructs, correlations between the two scales would not be so high (*r*≥0.80, Field, 2013) and the associations with other measures would appear in different patterns.

## Materials and Methods

A sample of 850 Korean adults completed an online survey. To recruit participants, we used an online research participants system, and proportional allocation was applied to readily represent various age (+18) and gender groups. The mean age of the sample was 43.98years (SD=13.77; range: 18–69years) and 50% were female (*n*=425). Only native Korean speakers were retained for the study. Participants accessed an online research participation system and provided informed consent. The anonymous survey was comprised of 198 questions, including demographic questions. There was no missing data, and all 850 participants completed all items on SOCS and other self-reported measures.

With the exception of SOCS, the following measures, which were expected to be theoretically related to compassion toward others and self, were used for assessing the validity of the scales.

### Sussex-Oxford Compassion Scale for Others and Sussex-Oxford Compassion for the Self

SOCS-O and SOCS-S are newly developed scales, for which psychometric properties were thoroughly examined and supported (Gu et al., 2020). The original versions of SOCS-O and SOCS-S were translated into Korean by the first author (JK) and back translated by a bilingual student majoring in psychology and who lived in an English-speaking country more than 15years, after the authors confirmed the accuracy of the translation (see Supplementary Material). Each scale was comprised of 20 items that assess compassion for the self and others, and participants are asked how true each statement is for them on a 5-point Likert scale ranging from 1 to 5.

### Five Facet Mindfulness Questionnaire

Five Facet Mindfulness Questionnaire (FFMQ) is a 15-item self-report questionnaire developed by Baer et al. (2006). The scale assesses the general tendency to be mindful in everyday life with 15 items that reflect five facets of mindfulness: observing; describing; acting with awareness; non-judging of inner experience; and non-reactivity to inner experience. For the current study, items assessing “observing” were excluded as it was expected that the current sample had little or no experience of practicing meditation. The Korean version of FFMQ-15 (Cheong et al., 2017) was used and the estimate of internal consistency for the current sample was 0.63.

### Self-Compassion Scale

SCS-12 is a short form of the original 26 items (Neff, 2003a). It consists of items that assess self-kindness, self-judgment, common humanity, isolation, mindfulness, and over-identification (Raes et al., 2011). The short form and the original form were found to have the same factor structure (Raes et al., 2011). Psychometric properties of the Korean version of SCS-12 were supported by Kim et al. (2008). Cronbach’s alpha for SCS-12 items in the current sample was 0.88.

### Compassion Love Scale

Shin and Choi (2013) translated and developed a short Korean version of CLS (Sprecher and Fehr, 2005). CLS-K11, for which psychometric properties are well supported, consists of 11 items. Items measure respondents’ tendency to be compassionate toward strangers and humankind at large. Participants’ responses on items were scored on a 7-point Likert scale ranging from 1 to 7. The estimate of internal consistency of this scale for the current sample was 0.94.

### Warwich-Edinburgh Mental Well-Being Scale

Warwich-Edinburgh Mental Well-Being Scale (WEMWBS) consists of 14 items and measures positive mental wellbeing (Stewart-Brown et al., 2009). The Korean translated version of WEMWBS was developed and its psychometric properties were supported by Kim et al. (2014). Responses were provided on a 5-point Likert scale ranging from 1 (never) to 5 (always). Cronbach’s alpha for WEMWBS for the current sample was 0.94.

### Interpersonal Reactivity Index

Interpersonal Reactivity Index (IRI) is a measure of dispositional empathy (Davis, 1980). The scale includes four subscales: perspective taking; fantasy; empathic concern; and personal distress. Kang et al. (2009) translated IRI into Korean and supported its psychometric properties. In the current study, the “fantasy” subscale was excluded as it was regarded to be unrelated to core aspects of compassion. Respondents scored whether each statement on the questionnaire readily represented them on a 5-point Likert Scale (from 1 to 5). The estimates of Cronbach’s alpha were 0.61 (perspective taking), 0.64 (empathic concern), and 0.76 (personal distress).

### Depression Anxiety Stress Scale

DASS-21 is a shortened version of Depression Anxiety Stress Scale (DASS). The scale consists of three subscales that measure core symptoms associated with depression, anxiety, and stress. Each subscale is comprised of seven items. On a 4-point Likert scale (from 0 to 3), participants were instructed to indicate whether they had experienced the presence of each symptom over the past week. For the current study, the Korean translation of the DASS-21 and the estimate of Cronbach’s alpha were 0.89 (stress), 0.90 (anxiety), and 0.92 (depression).

### Statistical Analyses

To examine the factor structure of SOCS-O and SOCS-S, confirmatory factor analysis was conducted with R 3.2.4 lavaan package (Rosseel, 2012). As Gu et al. (2020) supported the five-factor structure of SOCS-O and SOCS-S, we examined the five-factor correlated model and the five-factor hierarchical model. In the five-factor correlated model, items load on respective factors that represent the five-element definition of compassion, but within a five-factor hierarchical model, each factor loads on an overarching compassion factor (Strauss et al., 2016). Goodness of fit was tested with the following indices: the comparative fit index (CFI; Bentler, 1990); the root mean square error of approximation (RMSEA; Steiger, 1990); and the standardized root mean square residual (SRMR). Given the considerations of Bentler (1990), CFI greater than 0.90 was considered to indicate a good fit. In addition, following the suggestion of Browne and Cudeck (1993), RMSEA less than 0.05 was an indication of close fit, and a value between 0.08 and 0.10 indicates mediocre fit. An SRMR of between 0 and 0.05 indicates a good fit, and a value between 0.05 and 0.10 indicates an acceptable fit (Schermelleh-Engel et al., 2003). Following Gu et al. (2020), chi-square test of model of fit was reported, but not used as a fit index due to problems of hypersensitivity.

In order to examine whether total scale scores obtained differ in each gender group, independent *t*-tests were conducted. The internal consistency (Cronbach’s alpha) of the SOCS-O and SOCS-S was computed using SPSS version 25. Floor and ceiling effects were assessed by computing the percentage of respondents who scored 100 (highest score) or 0 (lowest score) on SOCS-O and SOCS-S. Following Terwee et al. (2007), the floor and ceiling effects of the scales were examined by calculating the percentage of respondents with the highest and lowest possible points. When less than 15% of the sample achieved the highest or lowest score, both scales were determined to capture response variability.

## Results

### Factor Structure of the SOCS

**Figure 1:**
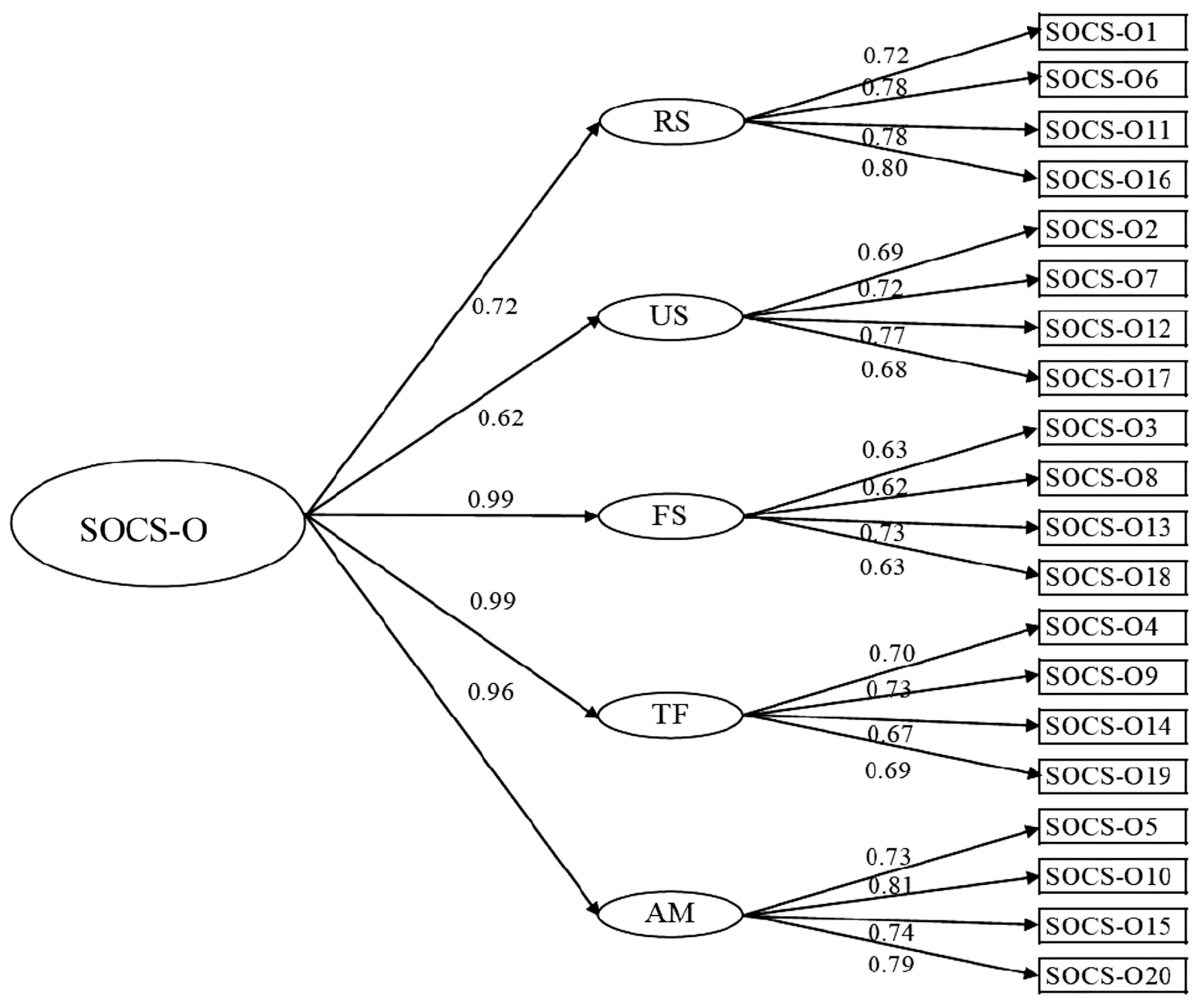
Hierarchical five-factor model of the SOCS-O. Straight arrows: standardized item loadings. SOCS-O, Sussex-Oxford Compassion Scale for Others; RS, recognizing suffering; US, understanding the universality of suffering; FS, feeling for the person suffering; TF, tolerating uncomfortable feelings; and AM, acting or being motivated to act to alleviate suffering.

**Figure 2:**
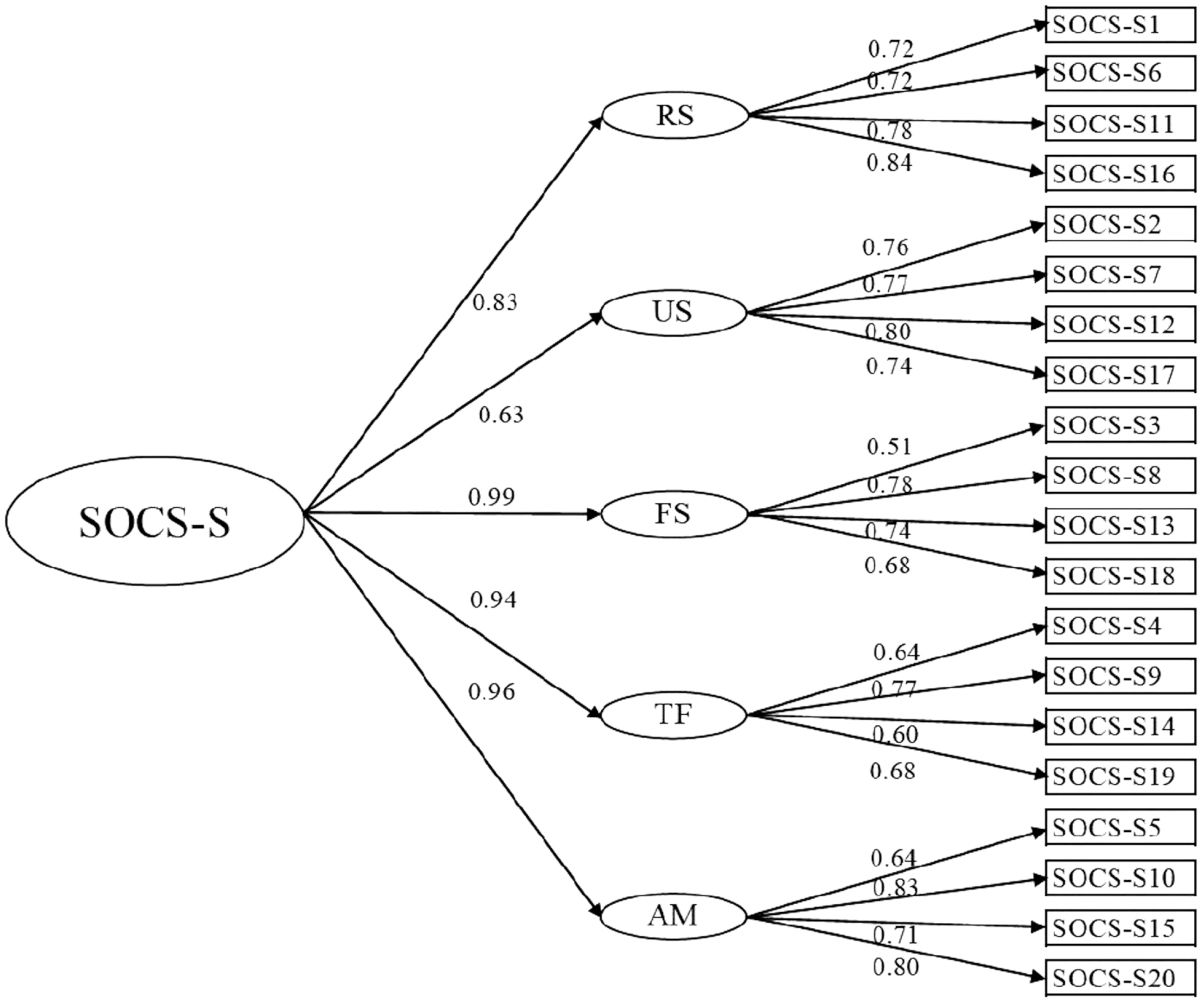
Hierarchical five-factor model of the SOCS-S. Straight arrows: standardized item loadings. SOCS-S, Sussex-Oxford Compassion Scale for the Self; RS, recognizing suffering; US, understanding the universality of suffering; FS, feeling for the person suffering; TF, tolerating uncomfortable feelings; and AM, acting or being motivated to act to alleviate suffering.

Most of the fit indices indicated good fit of the five-factor correlated models and the five-factor hierarchical model, and all item loadings in these two models were significant. According to fit indices and factor loadings, the five-factor hierarchical model appeared as best fitting the data for both SOCS-O [*X*^2^(165)=923.51, *p*<0.001; RMSEA=0.07; SRMR=0.06; CFI=0.91] and SOCS-S [*X*^2^(165)=942.13, *p*<0.001; RMSEA=0.07; SRMR=0.06; CFI=0.92]. Table 1 shows the fit indices for the five-factor correlated and the five-factor hierarchical CFA models for SOCS-O and SOCS-S. Figure 1 shows standardized item loadings into five latent factors and the overarching compassion factor in the five-factor hierarchical model for the SOCS-O, and Figure 2 shows standardized item loadings into factors in the five-factor hierarchical model for SOCS-S.

**Table 1:** Fit indices for compassion models tested.

Scale	Model	CFI	RMSEA (90% CI)	SRMR	*X* ^2^
Compassion for others	Five-factor correlated model	0.913	0.074 (0.06, 0.07)	0.056	923.508 (165)
Compassion for the self	Five-factor hierarchical model	0.916	0.074 (0.070, 0.079)	0.060	942.136 (165)

*CFI, comparative fit index; RMSEA, root mean square error of approximation; and SRMR, standardized root mean square*.

### Invariance Testing

To examine measurement invariance across the two gender groups, we conducted a multigroup confirmatory factor analysis. First, configural invariance models of SOCS-O and SOCS-S were tested for men and women. When the same hierarchical five-factor structure was specified for men and women simultaneously, the results indicated a good overall fit, suggesting that the equivalent factor structure of SOCS-O and SOCS-S holds up similarly for both gender groups, SOCS-O: *X*^2^(330)=1116.98, *p*<0.001; RMSEA=0.07; SRMR=0.06; CFI=0.91; SOCS-S: *X*^2^(330)=1165.60, *p*<0.001; RMSEA=0.08; SRMR=0.06; CFI=0.91. Second, to examine metric invariance, we constrained the factor loadings to be equivalent across male and female groups while allowing item intercepts to vary freely. Our analyses supported equivalent factor loadings, suggesting that the five factors of the SOCS-O and SOCS-S were assessed by respective items in a similar manner and with similar magnitude across the two gender groups, SOCS-O: $X_{\mathrm{diff}}^{2}$=32.92, Δ*df*=19, *p*=0.02; SOCS-S: $X_{\mathrm{diff}}^{2}$=41.56, Δ*df*=19, *p*=0.002. Third, we conducted scalar invariance testing to examine whether the item intercepts are equivalent for people of different genders. The analyses failed to support the intercept equivalence in both SOCS-O and SOCS-S, suggesting that one or more parameters were not equivalent across groups, SOCS-O: $X_{\mathrm{diff}}^{2}$=51.44, Δ*df*=14, *p*<0.001; SOCS-S: $X_{\mathrm{diff}}^{2}$=58.36, Δ*df*=14, *p*<0.001. When the intercepts of the items 8 and 19 were freely estimated, partial scalar invariance of SOCS-O could be established [*X*^2^(360)=1174.25, *p*=0.02; RMSEA=0.07; SRMR=0.06; CFI=0.91]. The estimates of intercepts of item 8 and 19 in the two groups were 4.06 (women)/3.91 (men) and 3.28 (women)/3.47 (men), respectively. Free estimation of the intercepts of item 14 and 19 of SOCS-S established partial invariance of SOCS-S established [*X*^2^(360)=1239.15, *p*=0.001; RMSEA=0.08; SRMR=0.07; CFI=0.91]. The estimates of intercepts of item 14 and 19 in the two groups were 3.34 (women)/3.20 (men) and 3.16 (women)/3.36 (men), respectively.

### Interpretability

To examine gender differences in the SOCS-O and SOCS-S scores, independent *t*-tests were conducted, and mean scores were compared. Contrary to the findings of Gu et al. (2020), females (M=72.54, SD=10.20, *n*=425) did not scored significantly higher on SOCS-O than with males (M=70.46, SD=10.27, *n*=425), *t*(848)=2.96, *p*=0.003. In contrast, there were no significant differences between males (M=72.21, SD=11.25, *n*=425) and females (M=73.28, SD=11.79, *n*=425) in SOCS-S scores, *t*(848)=1.35, *p*=0.176.

### Internal Consistency

The estimates of Cronbach’s alpha for total SOCS-O and subscale items ranged from 0.75 to 0.93, and for total SOCS-S and subscale items ranged from 0.76 to 0.94. Given the considerations of Kline (2000), these values were assessed to be adequate for measures of psychological constructs. Table 2 presents detailed values of Cronbach’s alpha for total SOCS-O and SOCS-S scales and subscale items.

**Table 2:** Cronbach’s alpha for SOCS-O and SOCS-S scale and subscale items.

	Compassion for others	Compassion for the self
Total scale	0.93	0.94
Recognizing suffering	0.85	0.85
Understanding the universality of suffering	0.80	0.85
Feeling for the person suffering	0.75	0.76
Tolerating uncomfortable feelings	0.79	0.76
Acting or being motivated to act to alleviate suffering	0.85	0.83

*SOCS-O, Sussex-Oxford Compassion for Others scale; SOCS-S, Sussex-Oxford Compassion for the Self scale*.

### Floor and Ceiling Effects

None of the participants obtained the lowest possible score (0) on SOCS-O and SOCS-S, and 0 and 0.7% of participants scored the highest possible score (100) on SOCS-O and SOCS-S, respectively. Since less than 15% of the sample received extreme scores, both scales were assessed to capture response variability.

### Convergent and Discriminant Validity

Convergent validity is the degree to which a measure is related to constructs that are purported to be associated. Discriminant validity, in contrast, refers to the extent to which a measure does not measure unrelated constructs. In this respect, positive correlations between the SOCS scales and related constructs would indicate convergent validity, while negative correlations between the scales and unrelated constructs would indicate discriminant validity. The results of correlations between SOCS-O, SOCS-S, and other self-report measures are provided in Table 3. As expected, higher level of compassion for others was positively and significantly associated with compassionate love toward others at *r*≥0.50. Also, SOCS-O had a significant and large correlation (*r*≥0.50.) with the empathic concern and perspective taking subscales of the IRI in expected directions. Moderate to large correlations were found between SOCS-S and SCS, FFMQ, WEMWBS, and negative correlations were found between SOCS-S and the personal distress subscale of the IRI and the three subscales of the DASS. Although the relationships between SOCS-S and the subscales of the IRI and DASS were significant, the correlation values were rather small. The observed positive and significant correlations between the two scales of SOCS and other measures provided evidence for convergent validity and the negative relationships supported discriminant validity.

**Table 3:** Correlation coefficients between total scores on the SOCS-O and SOCS-S and other self-report measures.

	FFMQ	SCS	CLS-K11	WEMWBS	IRI-EC	IRI-PT	IRI-PD	DASS-S	DASS-A	DASS-D
SOCS-O	0.25^**^	0.19^**^	0.56^**^	0.44^**^	0.50^**^	0.50^**^	−0.10^**^	−0.07^*^	−0.11^**^	−0.14^**^
RS	0.21^**^	0.12^**^	0.43^**^	0.37^**^	0.32^**^	0.36^**^	−0.11^**^	0.01	−0.01	−0.06
US	0.21^**^	0.15^**^	0.20^**^	0.28^**^	0.33^**^	0.36^**^	−0.06	−0.10^**^	−0.19^**^	−0.15^**^
FS	0.16^**^	0.14^**^	0.54^**^	0.36^**^	0.54^**^	0.47^**^	−0.01	−0.04	−0.08^*^	−0.10^**^
TF	0.24^**^	0.20^**^	0.54^**^	0.39^**^	0.44^**^	0.47^**^	−0.12^**^	−0.11^**^	−0.11^**^	−0.13^**^
AM	0.21^**^	0.16^**^	0.60^**^	0.40^**^	0.44^**^	0.41^**^	−0.10^**^	−0.01	−0.06	−0.12^**^
SOCS-S	0.49^*^	0.48^**^	0.35^**^	0.54^**^	0.33^**^	0.38^**^	−0.25^**^	−0.22^**^	−0.24^**^	−0.28^**^
RS	0.39^**^	0.32^**^	0.23^**^	0.40^**^	0.28^**^	0.30^**^	−0.19^**^	−0.15^**^	−0.19^**^	−0.20^**^
US	0.27^**^	0.24^**^	0.18^**^	0.32^**^	0.32^**^	0.36^**^	−0.09^**^	−0.15^**^	−0.25^**^	−0.20^**^
FS	0.37^**^	0.39^**^	0.36^**^	0.47^**^	0.28^**^	0.30^**^	−0.17^**^	−0.15^**^	−0.14^**^	−0.20^**^
TF	0.49^**^	0.49^**^	0.37^**^	0.52^**^	0.22^**^	0.30^**^	−0.28^**^	−0.21^**^	−0.15^**^	−0.21^**^
AM	0.50^**^	0.55^**^	0.34^**^	0.57^**^	0.26^**^	0.31^**^	−0.29^**^	−0.27^**^	−0.26^**^	−0.34^**^

*SOCS-O, Sussex-Oxford Compassion for Others; SOCS-S, Sussex-Oxford Compassion for the Self; RS, recognizing suffering, US, understanding the universality of suffering, FS, feeling for the person suffering, TF, tolerating uncomfortable feelings, AM, acting or motivation to act to alleviate suffering; FFMQ, Five-Factor Mindfulness Questionnaire; SCS, Self-Compassion Scale; CLS-K11, Compassionate Love Scale; WEMWBS, Warwich-Edinbuirgh Mental Well-Being Scale; IRI-EC, Interpersonal Reactivity Index (Empathetic Concern subscale); IRI-PT, Interpersonal Reactivity Index (Perspective Taking subscale); IRI-PD, Interpersonal Reactivity Index (Personal Distress subscale); DASS-S, Depression, Anxiety, and Stress Scale (Stress subscale); DASS-A, Depression, Anxiety, and Stress Scale (Anxiety subscale); and DASS-D, Depression, Anxiety, and Stress Scale (Depression subscale)*.

* *p<0.05 and*

** *p<0.01*.

## Discussion

The aim of the current research was to develop a Korean version of SOCS and evaluate the psychometric properties of two measures assessing compassion toward the self and others: the SOCS-S and the SOCS-O. Consistent with the previous study conducted by Gu et al. (2020), the five-factor hierarchical structure achieved satisfactory model fit for both scales. This supports that the relationships between the five elements of compassion directed toward others and the self are similarly found and operationalized in Korean culture as well. In addition, this study aimed to examine whether the factor structure differs in relation to gender by exploring measurement invariance. First, for the SOCS-O scale, we were able to establish full metric invariance. However, we failed to establish full scalar invariance, but partial scalar invariance with free estimation of item 8 (“When I hear about bad things happening to other people, I feel concern for their well-being”) and 19 (“When someone else is upset, I can be there for them without feeling overwhelmed by their distress”). The intercept of item 8 was higher among women and a higher intercept of item 19 was exhibited among men.

Several explanations can address the discordance. First, there is an empirical evidence for gender differences in sympathy, which is defined as a response of concern for a person experiencing emotional distress (Gruen and Mendelsohn, 1986). According to Strauss et al. (2016), sympathy is a related construct of compassion as the latter includes recognizing and emotionally connecting to another person’s suffering as core components. It appears that whereas for men to feel sympathy, both knowing and feeling for the person’s pain are required, women can feel sympathy when they know that someone is in pain (Goldstein and Winner, 2012). That is to say that for women’s sympathy appears more others directed, and it could be elicited without necessarily having to connect to the other person’s suffering. In contrast, men’s sympathy is more self-directed and both knowing and feeling another’s distress are required (Goldstein and Winner, 2012). These differences in sympathetic responses may have been reflected in observed differences in baseline scores of items 8 and 19, which assess emotional connectedness to another’s emotional distress and the capacity to tolerate uncomfortable feelings.

Scalar invariance of SOCS-S also failed, but partial scalar invariance was marginally established with the free estimation of item14 (“I connect with my own suffering without judging myself”) and 19 (“When I’m upset, I can let the emotions be there without feeling overwhelmed”). The intercepts of item 14 were higher among women while higher intercepts of item 19 were observed among men. Potential differences in mindfulness among men and women could account for these findings. Self-compassionate individuals exhibit mindful awareness of their negative thoughts and emotions and approach them with a balanced view without judgment (Bishop et al., 2004; Neff and Dahm, 2015). A study conducted by Alispahic and Hasanbegovic-Anic (2017) has reported that while women display greater levels of mindfulness, significant gender differences exist. It appears that women in general are better at noticing their emotions, whereas men had a greater tendency to attend to what is happening in the present moment. Moreover, the tendency of men to experience less intense emotions (Diener et al., 1985) may have been reflected in the score differences seen in item 14 and 19.

Our independent *t*-tests revealed that women scored significantly higher on the SOCS-O compared to men while there were no significant differences between male and female participants’ scores on SOCS-S. Consistent with our findings, results from Gu et al. (2020) and previous studies (e.g., Sprecher and Fehr, 2005; Burnell and Agan, 2013; Martins et al., 2013) measuring compassion for others with different tools have shown higher levels of compassion among women. The difference in the ability to recognize and precisely decode emotions could provide an explanation for these outcomes. Indeed, an abundance of research has demonstrated that women better identify the emotions of others compared to men by a small to modest magnitude (e.g., Rotter and Rotter, 1988; Hoffmann et al., 2010; Connolly et al., 2019). If women are in fact better at recognizing and decoding emotions, this can facilitate the experience of compassion for others going through difficult times. For the SOCS-S scale, we did not find evidence for gender differences. In fact, results from previous research on gender differences in self-compassion have been inconsistent. Similar to our findings, some studies have found no gender differences in self-compassion (Raque-Bogdan et al., 2011; Neff and Pommier, 2013), while others have found significant differences in relation to gender, demonstrating lower levels of self-compassion in women compared to men (Neff and McGehee, 2010; Yarnell and Neff, 2013; Yarnell et al., 2015).

Our comparison of women and men’s self-compassion scores did not support significant gender differences. In explaining this finding, the distinctive cultural features of Korean society should be taken into consideration. First, this finding may be attributable to Korean men’s adherence to masculinity. In a recent cross-cultural study, South Korean society has been categorized as having high masculine cultural orientation (Montero-Marin et al., 2018). This adherence to traditional masculine norms of being strong and unemotional might result in inhibition from vulnerable feelings and impair individuals’ ability to be understanding toward themselves without judging themselves negatively in times of need (Levant et al., 2009). While further investigation is required, strong adherence to cultural orientation of masculinity at the society level may blur the gender differences in self-compassion among Korean men and women. Furthermore, according to Hofstede’s cultural value dimensions, South Korean society has been categorized as having high collectivism, large power distance, long-term orientation, and less tolerance to uncertainty (Hofstede and Hofstede, 2005). This implies that Korean society is oriented toward collectivistic obligations and bonds, vertically stratified authority, future-oriented values (e.g., perseverance), and imposing more rules and standards on individuals (Kim and Kim, 2009). Although further research is required, these characteristics may contribute to distinctive patterns and degrees of gender differences in self-compassion, compared to countries with different cultural values (Montero-Marin et al., 2018).

Consistent with the predictions, the SOCS-O scale showed large correlations with CLS-K11, the measure of compassionate love toward others, and two subscales of IRI, assessing empathetic concern and perspective taking. We also found small to moderate significant correlations between the SOCS-O and mindfulness, wellbeing, and mental health problems. These results were consistent with the findings of Gu et al. (2020), which demonstrated significant relationships between compassion toward others and mindfulness, wellbeing, and mental health problems, in contrast with previous research which showed no relationship between compassion toward others and these variables (e.g., López et al., 2018). As hypothesized, the SOCS-S showed significant positive correlation with the SCS. In line with the prediction, the SOCS-S had significant correlations in the expected directions with measures of mindfulness, wellbeing, stress, anxiety, and depression. Although both the SOCS-O and SOCS-S showed expected correlations with mindfulness, wellbeing, stress, and anxiety, they differed in terms of their patterns of associations with DASS, the measure of stress, anxiety, and depression. Whereas all five subscales of the SOCS-S showed significant negative associations with DASS, correlations between the “recognizing suffering” subscale of the SOCS-O and stress, anxiety, and depression were not significant. In addition, the “acting or being motivated to act to alleviate suffering” subscale was significantly correlated only with depression. One possible explanation for these findings is the relatively more powerful influence of self-compassion than compassion toward others on one’s mental health status. Indeed, a number of studies have demonstrated the potent impact of self-compassion on the psychological health of Koreans (e.g., Lee and Bang, 2010; Kyeong, 2013; Joeng et al., 2017), and our findings may provide support for the need for effective self-compassion enhancement interventions for both non-clinical and clinical populations. The correlations between the SOCS-O and SOCS-S were large (*r*=0.65), but not so large to suggest that they measure two distinguished constructs. In addition, whereas the SOCS-S had a moderate correlation with the Self-Compassion Scale (SCS), the association between the SOCS-O and SCS was small, indicating compassion toward others and the self are not redundant. Taken together, our correlation analyses also showed evidence of adequate convergent and discriminant validity of the SOCS-O and SOCS-S. Also, the internal consistency of total SOCS-O and SOCS-S scale and subscales was satisfactory, and the scales exhibited no evidence of floor and ceiling effects, demonstrating the SOCS is a reliable and valid tool for measuring compassion toward others and self-compassion.

### Limitations

There are several limitations to consider. In investigating the psychometric properties of the SOCS, Gu et al. (2020) used two independent samples consisting of healthcare staff and undergraduate students and examined whether their scores on both scales differ in relation to meditation experience, level of education, and marital status. However, our sample included the general population who completed the anonymous online survey, and we did not collect the sample’s demographic information other than age and sex. Therefore, it would be valuable to test different patterns of compassion toward others and the self in relation to theses variables. In particular, considering that a number of compassion-based interventions employ meditation to cultivate compassion (e.g., Cognitively Based Compassion Training; Compassion Cultivation Training; and Mindful Self-Compassion), whether the experience of meditation has a significant effect on SOCS-O and SOCS-S scores should be examined by further research. Second, internal consistency of measures assessing mindfulness (FFMQ), perspective taking (IRI-PT), and empathetic concern (IRI-EC) was unsatisfactory (*α*<0.70, Kline, 2000). Hence, with respect to the convergent/discriminant validity of the SOCS, our findings should be interpreted carefully, and further research needs to re-investigate whether a convergent/discriminant validity is established using tools with adequate internal consistency. Lastly, we conducted a cross-sectional study and did not examine the temporal stability of the SOCS-O and SOCS-S. Hence, further research is required to examine the test–retest reliability of the scale.

## Conclusion

Our study was the first study that has examined the psychometric properties of a Korean version of the SOCS, the newly developed scale for measuring compassion directed to others and the self. Further, the large sample size and equal distribution of age and gender group applied to our sampling process support the validity and reliability of our findings. Thus, we conclude that the Korean version of the SOCS could be used as a promising instrument that comprehensively captures compassion with robust psychometric properties.

## Data Availability Statement

The datasets presented in this article are not readily available because all data are treated with complete confidentiality. Requests to access the datasets should be directed to J-WS, jwseo@jbnu.ac.kr.

## Ethics Statement

The studies involving human participants were reviewed and approved by Jeonbuk National University–Institutional Review Board. The participants provided their written informed consent to participate in this study.

## Author Contributions

JK wrote the first draft of the manuscript. J-WS provided the opinions and revised it critically. JK and J-WS revised the final manuscript. All authors contributed to the article and approved the submitted version.

## Funding

The research received funding from the Brain Korea 21 fourth project of the Korea Research Foundation (Jeonbuk National University, Psychology Department no. 4199990714213).

## Conflict of Interest

The authors declare that the research was conducted in the absence of any commercial or financial relationships that could be construed as a potential conflict of interest.

## Publisher’s Note

All claims expressed in this article are solely those of the authors and do not necessarily represent those of their affiliated organizations, or those of the publisher, the editors and the reviewers. Any product that may be evaluated in this article, or claim that may be made by its manufacturer, is not guaranteed or endorsed by the publisher.
