# Supplementary material for: Assessing Compassion in Korean Population: Psychometric Properties of the Korean Version of Sussex-Oxford Compassion Scales
Source: Front Psychol. 2021 Oct 11;12:744481. doi: 10.3389/fpsyg.2021.744481 (PMC8544640; doi:10.3389/fpsyg.2021.744481)
Supplement: Supplementary file 1 [file Table_1.DOCX]

Supplementary Material

# Supplementary Table 1: The Korean version of the SOCS-O

*다음 문항을 잘 읽고, 문항 내용이 자신과 어느 정도 일치하는 지를 체크 해 주십시오.

|  | 문항 | 전혀  아니다 | 거의  아니다 | 가끔  그렇다 | 종종  그렇다 | 항상  그렇다 |
| --- | --- | --- | --- | --- | --- | --- |
| 1 | 나는 타인들이 괴로움을 느낄 때, 그들이 내게 말해주지 않더라도 알아차린다. |  |  |  |  |  |
| 2 | 나는 모든 사람들이 삶의 어느 시점에서 괴로움을 경험한다는 것을 이해한다 |  |  |  |  |  |
| 3 | 나는 타인이 힘든 시기를 겪고 있을 때, 그들에게 동정심을 느낀다 |  |  |  |  |  |
| 4 | 나는 타인이 속상해 할 때, 그의 감정을 피하기보다는 열린 마음을 가지고 들어주려고 한다. |  |  |  |  |  |
| 5 | 나는 타인들이 힘들어 할 때, 도움이 될 만한 것들을 하려고 한다. |  |  |  |  |  |
| 6 | 나는 타인들이 괴로워할 때 알아차린다. |  |  |  |  |  |
| 7 | 나는 때로 속상함을 느끼는 것은 인간 속성의 일부라는 것을 이해한다. |  |  |  |  |  |
| 8 | 나는 타인에게 나쁜 일이 일어났다는 소식을 들을 때, 그들이 괜찮을지 걱정된다. |  |  |  |  |  |
| 9 | 나는 타인들이 속상해 할 때, 하기 힘들더라도 함께 있어주고 그들의 말을 들어준다. |  |  |  |  |  |
| 10 | 나는 누군가 힘든 시기를 겪고 있을 때, 그를 돌봐주려고 한다 |  |  |  |  |  |
| 11 | 나는 타인들이 겪는 괴로움의 초기 신호를 빨리 알아차린다. |  |  |  |  |  |
| 12 | 나와 마찬가지로, 타인들도 인생에서 힘겨운 사건이나 상황을 경험한다는 것을 안다. |  |  |  |  |  |
| 13 | 나는 타인이 속상해 할 때, 그가 어떻게 느끼고 있는지 알아차리려고 한다 |  |  |  |  |  |
| 14 | 나는 타인들을 판단하지 않으면서 그들의 고통에 공감한다 |  |  |  |  |  |
| 15 | 나는 도움을 필요로 하는 타인을 볼 때, 그들에게 가장 필요한 것을 해주려고 한다. |  |  |  |  |  |
| 16 | 나는 타인들이 겪는 괴로움의 신호를 알아차린다. |  |  |  |  |  |
| 17 | 나는 모두가 부당한 대우를 받을 때 속상할 수 있다는 것을 안다. |  |  |  |  |  |
| 18 | 나는 타인의 괴로움에 민감하다 |  |  |  |  |  |
| 19 | 나는 타인이 속상해 할 때, 그들이 느끼는 괴로움에 압도됨을 느끼지 않고 그들을 위해 곁에 있어줄 수 있다 |  |  |  |  |  |
| 20 | 나는 누군가 속상해 하는 것을 볼 때, 최선을 다해 그를 돌본다. |  |  |  |  |  |

# Supplementary Table 2: The Korean version of the SOCS-S

*다음 문항을 잘 읽고, 문항 내용이 자신과 어느 정도 일치하는 지를 체크 해 주십시오.

|  | 문항 | 전혀  아니다 | 거의  아니다 | 가끔  그렇다 | 종종  그렇다 | 항상  그렇다 |
| --- | --- | --- | --- | --- | --- | --- |
| 1 | 나는 내가 괴로움을 느낄 때 잘 알아차린다. |  |  |  |  |  |
| 2 | 나는 모든 사람들이 삶의 어느 시점에서 괴로움을 경험한다는 것을 이해한다 |  |  |  |  |  |
| 3 | 내가 힘든 시기를 겪을 때, 내 자신에게 동정심을 느낀다 |  |  |  |  |  |
| 4 | 나는 속상할 때 내 감정을 피하기보다는 받아들이려고 한다 |  |  |  |  |  |
| 5 | 나는 괴로움을 느낄 때 비록 원인에 대해서는 아무것도 할 수 없을지라도 기분이 나아지도록 노력한다. |  |  |  |  |  |
| 6 | 나는 내가 괴로워 할 때 알아차린다. |  |  |  |  |  |
| 7 | 나는 때로 속상함을 느끼는 것은 인간 속성의 일부라는 것을 이해한다. |  |  |  |  |  |
| 8 | 나에게 나쁜 일이 일어날 때, 내 자신을 돌봐주고 싶은 마음이 든다. |  |  |  |  |  |
| 9 | 나는 나의 괴로움이 나를 압도하게 놔두지 않으면서 그것을 이해할 수 있다. |  |  |  |  |  |
| 10 | 나는 힘든 시기를 겪고 있을 때, 내 자신을 돌보려고 한다. |  |  |  |  |  |
| 11 | 나는 내가 겪는 괴로움의 초기 신호를 빨리 알아차린다. |  |  |  |  |  |
| 12 | 나와 마찬가지로, 타인들도 인생에서 힘겨운 사건이나 상황을 경험한다는 것을 안다. |  |  |  |  |  |
| 13 | 나는 속상함을 느낄 때, 내가 어떻게 느끼고 있는지 이해하려고 한다 |  |  |  |  |  |
| 14 | 나는 내 자신을 판단하지 않으면서 나의 고통을 인식한다 |  |  |  |  |  |
| 15 | 나는 속상할 때, 내 자신에게 가장 필요한 것을 하려고 한다 |  |  |  |  |  |
| 16 | 나는 내가 겪는 고통의 신호들을 알아차린다. |  |  |  |  |  |
| 17 | 살면서 상황이 좋지 않을 때면, 우리 모두가 괴로워할 수 있다는 것을 안다 |  |  |  |  |  |
| 18 | 내 자신에게 실망했을 때에도, 괴로움을 느낄 때면 내 자신에 대해서 따뜻한 마음을 느낄 수 있다. |  |  |  |  |  |
| 19 | 나는 속상할 때, 감정에 압도되지 않으면서 경험해 낼 수 있다 |  |  |  |  |  |
| 20 | 나는 속상할 때, 내 자신을 돌보기 위해 최선을 다한다. |  |  |  |  |  |
